# Signaling involved in neurite outgrowth of postnatally born subventricular zone neurons in vitro

**DOI:** 10.1186/1471-2202-11-18

**Published:** 2010-02-10

**Authors:** Konstantin Khodosevich, Hannah Monyer

**Affiliations:** 1 Department of Clinical Neurobiology, Interdisciplinary Center for Neuroscience, Im Neuenheimer Feld 364, 69120 Heidelberg, Germany

## Abstract

**Background:**

Neurite outgrowth is a key process during neuronal migration and differentiation. Complex intracellular signaling is involved in the initiation of neurite protrusion and subsequent elongation. Although, in general many constituents of the machinery involved in this multi-stage process are common for neurons in distinct brain areas, there are notable differences between specific neuronal subtypes.

**Results:**

We analyzed key intracellular components of neurite outgrowth signaling in postnatally born subventricular zone (SVZ) neurons *in vitro*. We showed that inhibitors of PI3K, Akt1, PKCζ and small GTPases significantly reduced neurite outgrowth. Transfection of SVZ-derived neurons with inactive forms of Rac1 or Cdc42 also decreased neurite length whereas transfection with constitutively active forms of Rac1, Cdc42 or Akt1 as well as with full-length PI3K or PKCζ increased neurite length. PI3K, Akt1 and PKCζ acted upstream of the small GTPases Rac1 and Cdc42, which in turn modulate lamellipodia formation of SVZ-derived neurons.

**Conclusion:**

We showed the involvement of PI3K/Akt1/PKCζ/Rac1/Cdc42 pathway in neurite outgrowth of postnatally born SVZ neurons.

## Background

Newly born migrating neuroblasts usually have one neurite, which they use for active migration from the site of origin to their destination site. A complex activity of receptors, cell adhesion molecules as well as attractants and repellents modulate intracellular machinery regulating outgrowth of neurites.

Many signaling molecules have been identified to be involved in neurite outgrowth, from membrane receptors to cytoskeleton constituents [1-3]. The tip of neurite, neural growth cone, is enriched in actin filaments as well as different filament remodeling and adapter proteins [2-4]. Intracellular kinases, such as MAPK, ERK and PI3K, regulate formation of actin filaments while small GTPases link kinase signaling to actin cytoskeleton machinery [5,6]. Distribution of different cell membrane and cytoplasm components determines the polarization of neuroblasts, and thus the directionality of migration [7]. Much of the analysis of neurite outgrowth machinery has been done in rat embryonic hippocampal culture (e.g., [8-11]). However, analysis in other systems does not always correspond to hippocampal culture and sometimes are even in contradiction with the results obtained in hippocampal culture. For example, activation of small GTPase Rac1 promotes neurite extension in rat hippocampal culture [8] while its activation decreases the longest neurite length of rat cortical culture [12] and its inhibition promotes neurite outgrowth in chick dorsal root ganglion neuronal culture [13]. Also, while activation of PI3K-Akt pathway in hippocampal culture induces neurite outgrowth [9,10], stimulation of this pathway can inhibit neurite outgrowth or have no effect in neuronal-like PC12 cell line [14,15]. Thus, intracellular signaling regulating neurite outgrowth varies among different neuronal cell types and has to be analyzed separately for each cell type.

The majority of neurons in mammalian brain are born and migrate to their destination site during embryonic development. There are, however, two postnatal brain regions that continue to produce neurons - subventricular zone of lateral ventricles (SVZ) and subgranular zone of hippocampus [16-18]. Postnatally generated SVZ neuroblasts migrate via the rostral migratory stream (RMS) to the olfactory bulb where they mature into distinct interneuron subtypes, namely granule and periglomerular cells. We recently described the generation of transgenic mice, in which EGFP is expressed in the entire RMS [19], and optimized a procedure for RNA isolation from *in vivo *fluorescent RMS neuroblasts [20]. Using transgenic mice with the clearly EGFP labeled RMS, we isolated neuroblasts from two distinct locations, one in the immediate vicinity of the SVZ (posterior RMS, pRMS), and the other more rostral, closer to the bulb (anterior RMS, aRMS) [21]. We showed that the majority of upregulated genes and pathways in cells from the aRMS are involved in neuroblast migration. However, different genes/pathways can affect various cellular processes involved in neuroblast migration, e.g. we found that GluA1 (AMPA receptor subunit 1) probably modulates neuroblast polarization while Vav3 (guanine nucleotide exchange factor) is needed for growth cone formation [21].

Using a neurite outgrowth culture assay, we analyzed several upregulated aRMS genes of the PI3K/Akt1/PKCζ/Rac1/Cdc42 pathway to establish their role for neurite outgrowth of postnatally generated SVZ/RMS neuroblasts. We found that activation of several proteins in this pathway enhanced neurite outgrowth while their inhibition decreased outgrowth. PI3K, Akt1 and PKCζ acted upstream of the small GTPases Rac1 and Cdc42, which in turn modulate lamellipodia formation and neurite elongation.

## Results

### Development of morphological neuronal characteristics for postnatal SVZ/RMS culture

**Figure 1:**
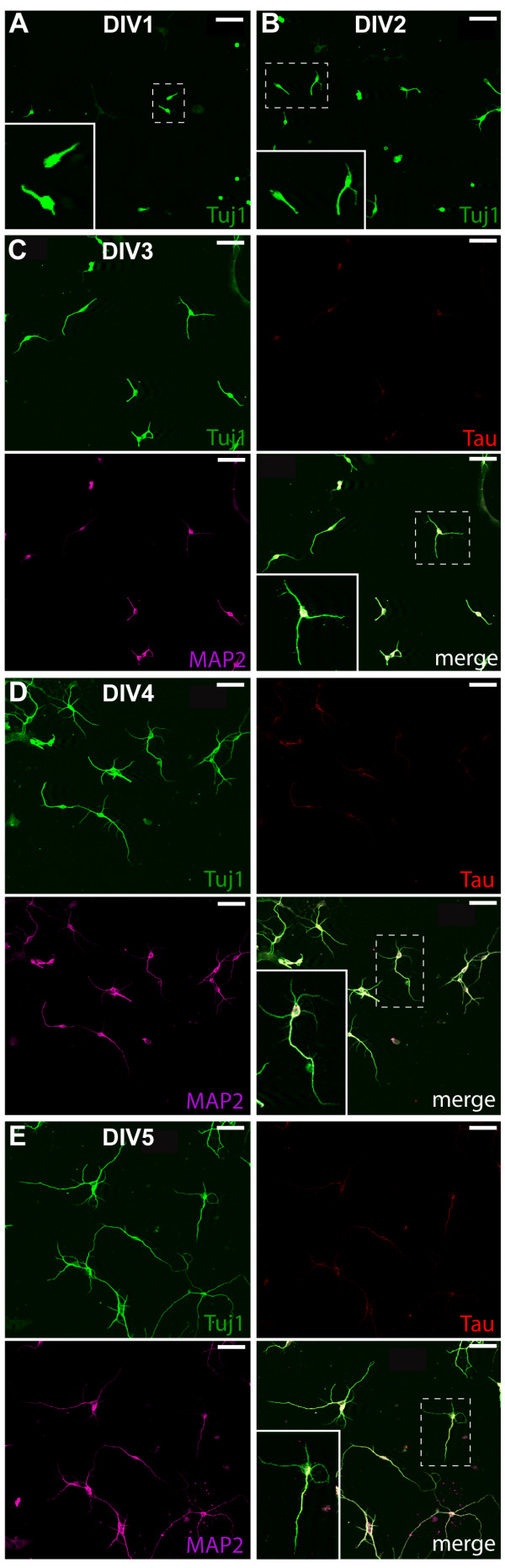
**Development of morphological characteristics in postnatal SVZ/RMS cultures**. (A) day 1 *in vitro *(DIV1). (B) DIV2. (C) DIV3. (D) DIV4. (E) DIV5. Scale bars are 50 μm.

We first established the development of different morphological characteristics in postnatal SVZ/RMS culture (Figure 1). At day 1 *in vitro *(DIV1), 70% of neurons already have one small neurite (Figure 1A) and at DIV2 many of them have 2 (32%) or 3 (21%) short neurites (Figure 1B). Thus, the initial neurite outgrowth for postnatal SVZ/RMS cultured neurons starts during the first day in culture and by the third day all neurons have at least 1 neurite. At DIV3, 90% of neurons have 2 or more neurites (Figure 1C), but all neurites of the cell are approximately of the same length, indicating that neurons are not polarized yet. At DIV4 and DIV5, 60% of neurons are polarized, i.e. have one major neurite longer than other neurites (Figure 1D, E). For analysis of polarization, it was not possible to use the standard dendritic and axonal markers MAP2 and Tau, respectively, (Figure 1C-E). Whilst most neurites exhibited MAP2 expression, only few polarized neurons (4%) were found to express Tau in their major neurite. This is not surprising, as it was shown that the major cell-type generated in the postnatal SVZ (granule cells) develop an axonless phenotype upon maturation [16,22].

### Neurite outgrowth analysis of SVZ/RMS-derived neurons following pharmacological treatment

We analyzed the involvement of different intracellular signaling molecules in neurite outgrowth of SVZ/RMS neuroblasts using protein inhibitors. For the analysis we chose 5 intracellular signaling molecules that are involved in neurite outgrowth/polarization of rat embryonic hippocampal cultures PI3K, Akt1, PKCζ, Rac1, Cdc42 [8-11]. Most of these genes are expressed in the postnatal SVZ and RMS at high levels and continue to be expressed in olfactory bulb according to the Allen Brain Atlas [23] (Additional file 1: Table S1). Expression of these genes in RMS was also shown in our previous microarray study [21].

**Figure 2:**
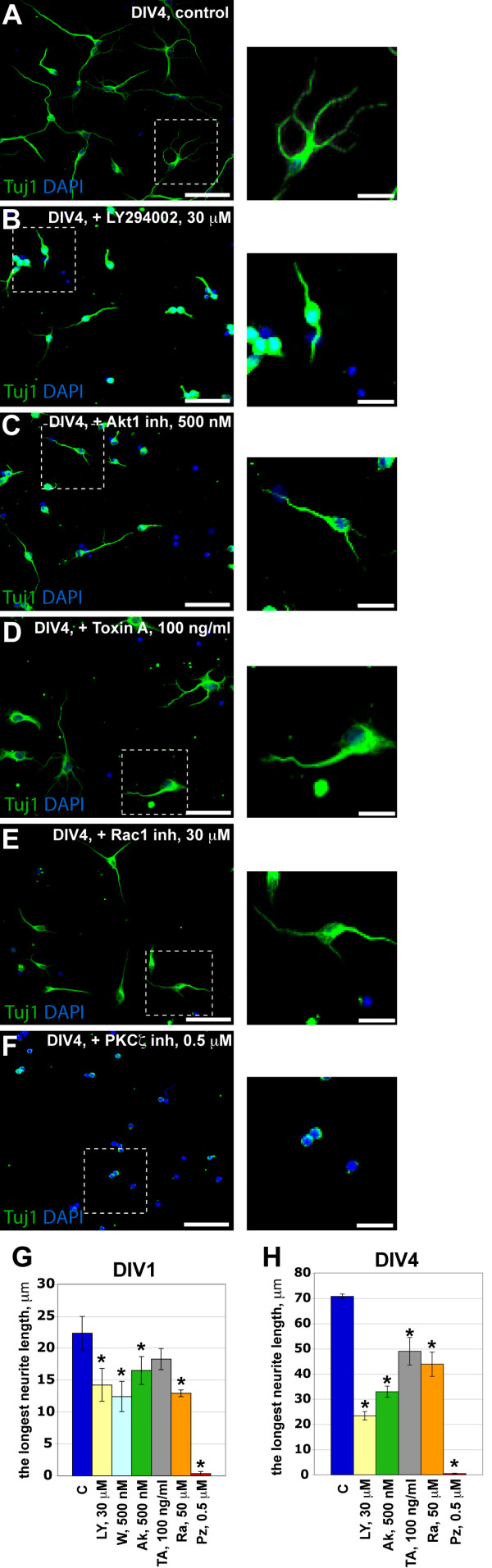
**Neurite outgrowth analysis of SVZ/RMS-derived neurons using pharmacological treatments**. (A) - (F) Examples of neurite outgrowth analysis in SVZ/RMS cultures treated by DMSO (A), PI3K inhibitor LY294002 (B), Akt1 inhibitor X (C), Toxin A of *C. difficile *(D), Rac1 inhibitor (E) and PKCζ pseudosubstrate inhibitor myristoylated (F) are shown. (G) and (H) All inhibitors decreased the longest neurite length of the Tuj1-positive cells at DIV1 and DIV4, respectively. (G), * - p < 0.05; (H), * - p < 0.001. Abbreviations: Ak - Akt1 inhibitor, C - control, LY - PI3K inhibitor LY294002, PZ - PKCζ inhibitor, Ra - Rac1 inhibitor, TA - Rho GTPases inhibitor Toxin A of *C. difficile*, Tuj1 - tubulin beta III to label immature neurons, W - PI3K inhibitor wortmannin. Scale bars in main illustrations are 50 μm and 20 μm in insets.

Dissociated SVZ/RMS neurons were cultured for one or four days in the presence or absence of different protein inhibitors following by fixation and staining with anti-Tuj1 (tubulin beta III - a marker for immature neurons) antibodies (number of experiments >= 3, cell number > 100). To be able to make correlations between the results obtained at DIV1 and DIV4, we used the length of the major neurite to assess neurite outgrowth, since at DIV1 the majority of neurons have only one neurite (Figure 1A). Inhibitors of PI3K (Wortmannin and LY294002), Akt1 (Akt1 inhibitor X) and PKCζ (PKCζ pseudosubstrate inhibitor myristoylated) as well as small GTPase inhibitors (*Clostridium difficile *Toxin A against all Rho GTPases and Rac1 inhibitor) significantly decreased neurite outgrowth after one day (p < 0.05, except *C. difficile *Toxin A) (Figure 2G). As vehicle we used DMSO. The inhibition of neurite outgrowth was more pronounced after four days of treatment (p < 0.001) (Figure 2A-F, H). Total neurite length was also decreased at DIV4 while the number of neurites was not changed (except for see PKCζ inhibitor, see Figure 2A-F). PI3K, Akt1 and PKCζ inhibitors had the strongest effect on neurite outgrowth. In fact, in dissociated cultures, SVZ/RMS neurons treated with low concentrations of PKCζ inhibitor (0.1-0.5 μM) failed to grow neurites longer than the cell body (Figure 2F-H). None of the inhibitors affected the initial neurite formation (i.e., number of neurons having one small neurite at DIV1), except of PKCζ inhibitor (data not shown). Many neurons still lacked a neurite even at DIV4 in PKCζ inhibitor-treated cultures (Figure 2F). Previously, PKCζ was shown to be involved in polarization, but not in neurite outgrowth of hippocampal culture neurons [11]. Our results possibly reveal a novel effect of PKCζ, demonstrating that in SVZ/RMS neurons PKCζ is not only important for cell polarization but also for neurite protrusion. The concentrations of the inhibitors used in the study did not decrease cell adhesion (Additional file 1: Figure S1A, B) and did not increase apoptosis following one- or four-day treatment in culture (Additional file 1: Figure S1C).

Treatment with Raf1 inhibitor or rapamycin (inhibitor of mTOR kinase) did not influence neurite outgrowth (data not shown).

### Neurite outgrowth analysis of SVZ/RMS-derived neurons following genetic manipulations

**Figure 3:**
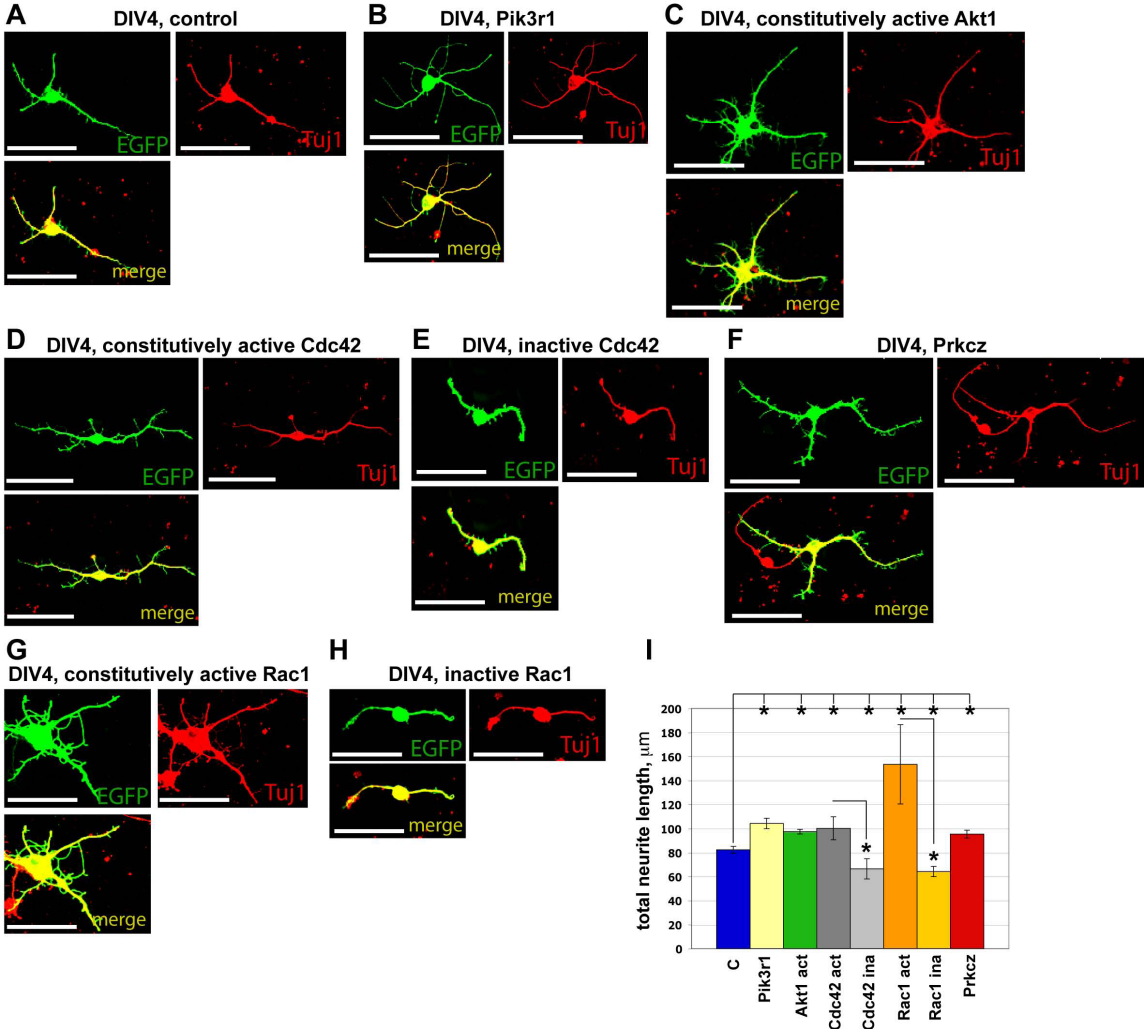
**Neurite outgrowth analysis of SVZ/RMS-derived neurons using genetic manipulations**. Cultured cells were transfected by both pEGFP and particular gene coding construct. (A) - (H) Examples of overexpression after transfections with control (A), Pik3r1 (B), constitutively active Akt1 (C), constitutively active Cdc42 (D), inactive Cdc42 (E), Prkcz (F), constitutively active Rac1 (G) and inactive Rac1 (H) constructs are shown. (I) Overexpression of constitutively active as well as full-length gene versions increased the total neurite length while inactive gene versions decreased it (* - p < 0.005). Abbreviations: Akt1 act - constitutively active form of Akt1, C - control (pCMV-SPORT6), Cdc42 act - constitutively active form of Cdc42, Cdc42 ina - inactive form of Cdc42, Pik3r1 - full-length Pik3r1 form, Prkcz - full-length PKCζ form, Rac1 act - constitutively active form of Rac1, Rac1 ina - inactive form of Rac1, Tuj1 - tubulin beta III to label immature neurons. Scale bars are 50 μm.

To strengthen the evidence for the involvement in neurite outgrowth of the intracellular molecules analyzed by protein inhibitors, we performed overexpression studies in dissociated SVZ/RMS neuronal cultures. Cultures were co-transfected with individual gene-expressing constructs together with an EGFP-expressing construct using lipofectamine 36 hours after plating, and the total length of green fluorescent cell neurites was measured 2.5 days after transfection (number of experiments >= 3, cell number > 100). Here we used the total length of neurites to assess neurite outgrowth, since after transfection with some overexpression constructs (especially with constitutively active gene versions), neurites grow in all directions. Thus, "total neurite length" is a more appropriate parameter than "major neurite length" to evaluate neurite outgrowth in transfection experiments. As a control we used pCMV-SPORT6 plasmid. Transfection with constitutively active Cdc42 and Rac1 expression constructs increased the total neurite length while inactive Cdc42 and Rac1 mutants decreased it (p < 0.005) (Figure 3A, D, E, G-I). Transfection with the activated Akt1 expression construct as well as with full-length PKCζ and Pik3r1 expression constructs also significantly (p < 0.005) increased neurite length (Figure 3A-C, F, I). Since overexpression studies were started at DIV2 while pharmacological studies at DIV0, they could affect cultures at different stages of maturation and, thus, have a slightly different effect. However, till DIV2 morphological changes in SVZ/RMS cultures were very subtle and noticeable neurite growth and development of polarization started between DIV3 and DIV4 (Figure 1). Hence, we believe that overexpression studies support the conclusions derived from the results obtained in pharmacological experiments.

### Involvement of small GTPase activation for neurite outgrowth of SVZ/RMS-derived neurons

**Figure 4:**
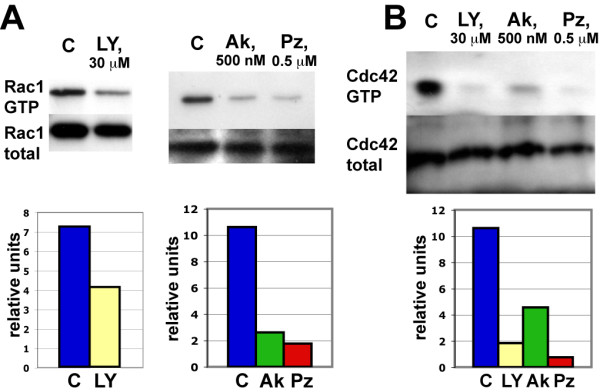
**Analysis of small GTPase activation in the SVZ/RMS neuronal culture**. (A) Rac1 and (B) Cdc42 pull-down assays. Cultured cells were treated with inhibitors to PI3K, Akt1 and PKCζ and the amount of activated Rac1-GTP and Cdc42-GTP forms were measured by Western-blot. Relative band intensities are shown under each western-blot. Abbreviations: Ak - Akt1 inhibitor, C - control, LY - PI3K inhibitor LY294002, PZ - PKCζ inhibitor.

Since activation of small GTPases modulates neurite outgrowth in different neuronal culture systems (reviewed in [24]), we tested the effect of PI3K, Akt1 and PKCζ inhibitors on the activation of GTPase Rac1 and Cdc42 in dissociated SVZ/RMS neuronal cultures. Following experiments were carried out in duplicates. Cultures were grown for 3 days in normal media followed by an additional culture day in the presence of PI3K, Akt1 or PKCζ inhibitors. There was a 1.8, 4 and 6-fold decrease of Rac1-GTP (activated Rac1 form) in PI3K, Akt1 and PKCζ inhibitor-treated cells, respectively (Figure 4A). Furthermore, application of PI3K, Akt1 and PKCζ inhibitors also decreased the amount of Cdc42-GTP (activated Cdc42 form) up to 8, 3.4 and 20-fold, respectively (Figure 4B).

### Phosphatidylinositols are involved in the polarization of SVZ/RMS-derived neurons

**Figure 5:**
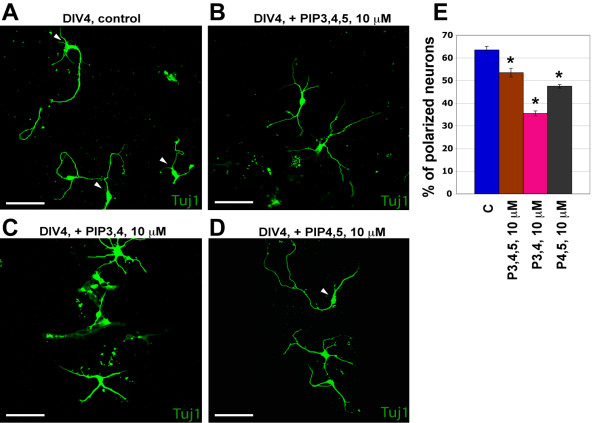
**Analysis of phosphatidylinositol (PIP) involvement in the polarization of SVZ/RMS-derived neurons**. Cultured cells were treated with different PIPs, and polarization of Tuj1-positive cells was determined at DIV4. (A) - (D) Examples of vehicle (A), PIP3,4,5 (B), PIP3,4 (C) and PIP4,5 (D) -treated SVZ/RMS cultures are shown. Arrowheads mark polarized neurons. (E) Treatment with PIPs significantly decreased number of polarized neurons in SVZ/RMS culture (* - p < 0.01). Abbreviations: Tuj1 - tubulin beta III to label immature neurons. Scale bars are 50 μm.

In culture, neuronal polarization occurs in several stages: extension of several undifferentiated neurites occurs after a few days followed by the more rapid growth of one neurite destined to become the axon (stage three) and the concomitant shortening of the neurites developing into dendrites [25]. Neurite outgrowth in hippocampal neuronal culture is known to be determined at least in part by the specific distribution of phosphatidylinositols (PIPs) on the cell membrane, and accumulation of PIP-(3,4,5)-P3 (PIP3,4,5) at the tip of neurites is important for their elongation [26]. Thus, we applied different PIPs - namely, PIP3,4,5, PIP-(3,4)-P2 (PIP3,4) and PIP-(4,5)-P2 (PIP4,5) - to dissociated SVZ/RMS neuronal cultures, and after 4 days of incubation we analyzed neuron polarization by staining with Tuj1 (we could not use MAP2/Tau expression due to the lack of Tau expression in the most of the cells, see above and Figure 1). Treatment with any of the tested PIPs caused a reduction (p < 0.01) in the number of neurons having only one long neurite (Figure 5A-E), whilst the total neurite length and neurite branching remained unchanged.

### Small GTPases are involved in lamellipodia formation of SVZ/RMS-derived neurons

**Figure 6:**
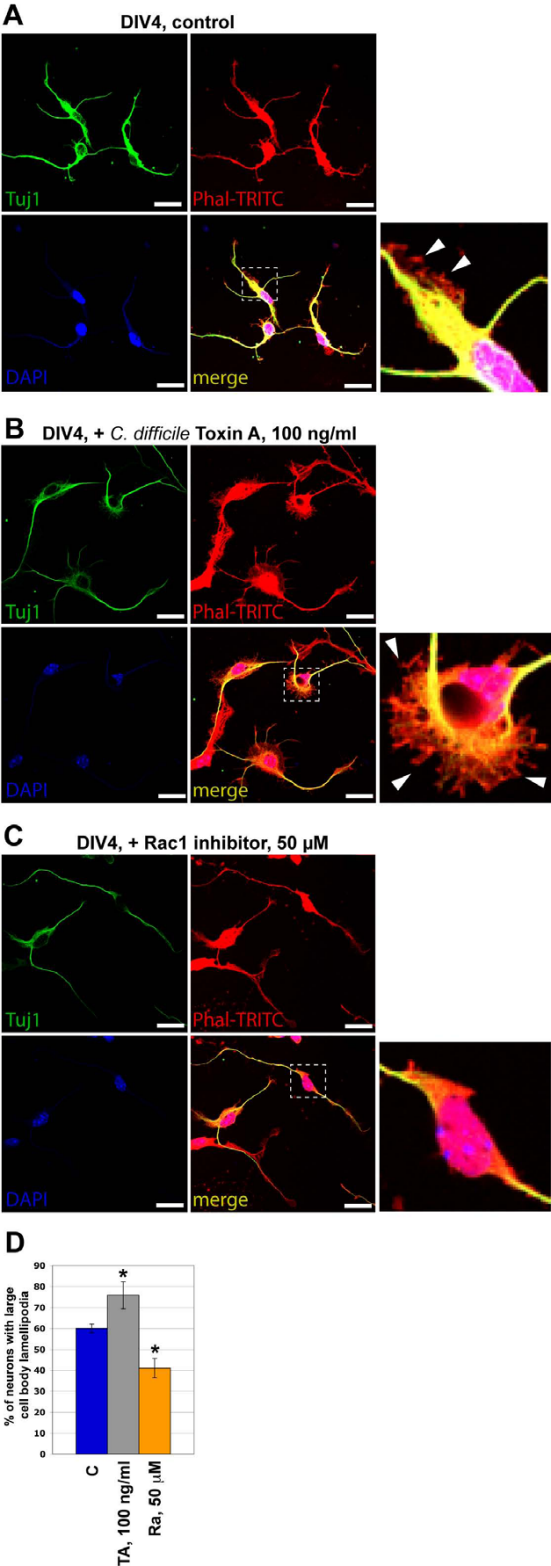
**Analysis of small GTPase involvement in lamellipodia formation of SVZ/RMS-derived neurons**. Lamellipodia outgrowth analysis in control (A) cultures and in cultures after *C. difficile *Toxin A and Rac1 inhibitor treatment (B) and (C), respectively. Arrowheads in insets mark large lamellipodia. Cultured cells were treated with inhibitors and the amount of neurons having large lamellipodia on cell body and major cell processes was calculated at DIV4. (D) While Rac1 inhibitor significantly decreased the number of neurons with large lamellipodia, Toxin A increased it (* - p < 0.001). Abbreviations: Phal-TRITC - phalloidin-TRITC complex to label F-actin, Ra - Rac1 inhibitor, TA - Rho GTPases inhibitor Toxin A of *C. difficile*, Tuj1 - tubulin beta III to label immature neurons. Scale bars are 20 μm.

An essential step in cell migration is lamellipodia propagation. It was shown for different cell types that the small GTPases Cdc42, Rac1 and Rho are involved in lamellipodia propagation by affecting/regulating actin scaffold proteins - Wasp/Wave [27]. We tested the influence of Rac1 inhibitor as well as *C. difficile *protein Toxin A, an inhibitor of Rho family small GTPases (Rac, Cdc42 and Rho) on lamellipodia formation around the neuronal cell body and the main neurite of SVZ/RMS neurons. We found that Rac1 inhibitor significantly decreased the number of neurons with large lamellipodia whereas Toxin A increased it (Figure 6A-D). The effect of Rac1 on lamellipodia formation most likely involves the activation of actin scaffold protein Wave1, as previously suggested [28].

## Discussion

**Figure 7:**
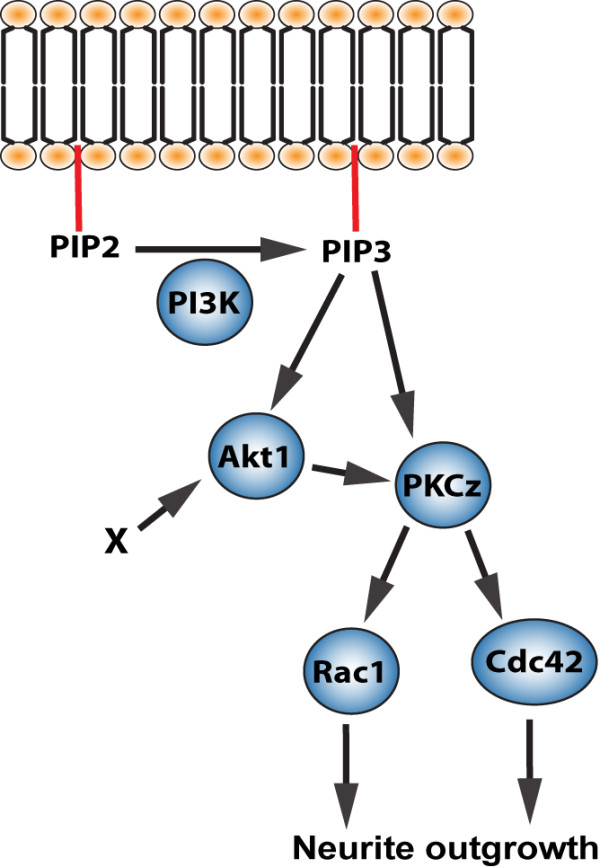
**Proposed model of neurite outgrowth pathway in postnatal SVZ/RMS-derived neurons**. The PI3K catalyzes phosphorylation of PIP4,5 (PIP2) to produce PIP3,4,5 (PIP3), which in turn activates Akt1 and PKCζ. It is likely that Cdc42 is preferentially activated directly via PIP3 to PKCζ and for Rac1 preferential activation is via PIP3,4,5 and Akt1 to PKCζ. Akt1 can be also activated by other kinases.

*In vitro *neurite outgrowth studies have frequently employed rat embryonic hippocampal cultures (e.g., [8-11]). However, in other culture systems results do not always correspond to those obtained in hippocampal culture. For instance, in contrast to hippocampal cultures, activation of the small GTPase Rac1 decreases the length of the longest neurite in rat cortical cultures [12] and its inhibition promotes neurite outgrowth in chick dorsal root ganglion neuronal cultures [13]. Also, stimulation of the PI3K/Akt pathway inhibits neurite outgrowth in neuronal-like PC12 cell line [14,15], whereas activation of this pathway in hippocampal cultures enhances neurite outgrowth [9,10]. Our work is the first study investigating neurite outgrowth in postnatally generated neurons. Although the results described here indicate that by and large the signaling pathway for neurite outgrowth in cultured postnatally born SVZ/RMS neurons is similar to that reported for rat hippocampal cultures or other neuronal cultures, there are some features that are specific for SVZ/RMS neuroblasts (Figure 7). PKCζ was previously shown to be involved in the regulation of neuronal polarization of several neuronal cell types in culture (e.g. [11,29]). However, treatment of SVZ/RMS culture with specific PKCζ inhibitor completely abolished neurite outgrowth - both, at DIV1 and DIV4 only few neurons had visible neurites. Since the decrease in neurite length is dramatic already at DIV1 when neurons are not polarized yet, we attribute the effect of PKCζ inhibitor treatment to an impairment of neurite outgrowth. Even very low doses of the PKCζ inhibitor (up to 0.1 μM) abrogated neurite outgrowth of SVZ/RMS neurons. Although much higher concentration of PKCζ inhibitor (10 μM) also decreased neurite length in rat hippocampal [11] and enteric [29] neuronal cultures, the effect was attributed specifically to an impairment in neuronal polarization, rather than neurite outgrowth.

Inhibition of PKCζ caused a stronger reduction in Rac1 and Cdc42 activation than inhibition of PI3K or Akt1 (Figure 4). This may be indicative of PKCζ acting downstream of PI3K and/or Akt1. The interaction of PKCζ with Cdc42 and Rac1 has been shown before [30-32], but differently from our results, Cdc42 and Rac1 acted upstream of PKCζ. In SVZ/RMS cultured neurons PKCζ can be envisaged to stabilize activated (GTP-bound) forms of Rac1 and Cdc42. Activation of both Rac1 and Cdc42 depended on PI3K/Akt1 activity (Figure 4). PI3K inhibitor reduced Cdc42 activation more than the Akt1 inhibitor, while reduction in GTP-bound Rac1 was stronger after application of the Akt1 inhibitor than the PI3K inhibitor. A possible scenario accounting for this findings may be that Cdc42 is activated downstream of PIP3 via PKCζ signaling, whilst activation of Rac1 involves a stronger recruitment of Akt1 (Figure 7). However, further experiments are required to directly prove the proposed model shown in Figure 7. Thus, it remains to be established whether activated versions of Rac1 and Cdc42 can rescue the phenotype caused by PI3K, Akt1 or PKCζ inhibitor. Conversely, activated forms of PI3K/Akt1/PKCζ should not have an effect on neurite outgrowth upon Cdc42 and Rac1 inhibition according to this model.

PI3K and Akt1 have been shown to be involved in neurite outgrowth in primary cultured neurons [11,33]. In hippocampal cultures, the accumulation of PIP3,4,5, the main enzymatic product of PI3K, specifies the future axon. PIP3,4,5 is required for axon elongation, and the specific distribution of PIPs in developing neurons is necessary for neuronal polarization [11,34]. Also for postnatal SVZ-derived neurons PI3K and Akt1 are required for neurite elongation as demonstrated in this study. However, affecting PIP3,4,5 distribution on cell membrane did not modify significantly neurite outgrowth while disturbing neuronal polarization. We hypothesize that for postnatal SVZ/RMS neurons PI3K activity is more important for neurite outgrowth while overall PIP distribution (which depends not only on PI3K, but also on many other proteins) is more important for neuronal polarization.

In contrast to the phenotype of postnatally generated dentate gyrus granule cells that grow dendrites and axons upon maturation, the majority of SVZ-generated neurons develop into axonless cells [16,22]. Indeed, we found that only few polarized neurons exhibited Tau expression (Figure 1). The results were confirmed using anti-Tau antibodies from different suppliers. We propose that the longest neurite of the polarized neurons in SVZ/RMS cultures corresponds to the major dendrite of granule cells, the main subtype of neurons produced in the SVZ [16,22].

In our lamellipodia analysis experiments we found that *C. difficile *protein Toxin A treatment resulted in formation of large lamellipodia around cell bodies of the neurons. Toxin A inhibits the activity not only of lamellipodia-regulating small GTPases, but also of the small GTPase Rho that stabilizes focal adhesion [35]. The observed phenotype could be the consequence of Rho inhibition that impaired the stabilization of focal adhesion and, as a result, promoted the formation of large lamellipodia.

It will be interesting to see whether other intracellular molecules that were shown to be involved in neurite outgrowth of postnatally born SVZ neurons can also modify PI3K/Akt1/PKCζ/Rac1/Cdc42 pathway signaling. One prominent candidate is PTEN, which negatively regulates PIP3 generation and PI3K signaling, thus, inhibiting neuronal polarization [11,36]. Since we showed the importance of PIP distribution for polarization of the SVZ/RMS neurons *in vitro*, the balance between PTEN/PI3K signaling may be important for proper neurite development. Another potentially interesting candidate is GSK3beta that was shown to inhibit axonal formation [36,37]. PI3K/Akt1/PKCζ/Rac1/Cdc42 pathway signaling may be also affected by some extracellular cues such as trophic factors and repellents/attractants. For instance, semaphorin [38] and netrin [39] signaling affect neurite outgrowth in vivo, and BDNF was shown to promote axonal differentiation *in vitro *via LKB1/Strad [40].

Neurite outgrowth is a fundamental neuronal feature and plays an important role in neuronal development during embryogenesis and in the adult brain. Although in general the machinery for neurite outgrowth has many common constituents when comparing various neuronal cell types, there are differences as shown here that need to be studied to better understand neuronal functions at the cellular level.

## Conclusion

In this study we analyzed intracellular signaling constituents involved in neurite outgrowth of postnatally born SVZ neurons. We showed that inhibition of PI3K, Akt1, PKCζ and small GTPases Rac1 and Cdc42 decreased neurite outgrowth. Since inhibition of PI3K, Akt1 and PKCζ resulted in a reduction of activated forms of Rac1 and Cdc42, we propose a model according to which the PI3K/Akt1/PKCζ cascade leads to the activation of the small GTPases Rac1 and Cdc42, thereby modulating the cytoskeleton machinery during neurite outgrowth (Figure 7).

## Methods

### Animals

For our experiments we used wild-type C57Bl/6 mice. All procedures with animals were performed according to the guidance of Heidelberg University Animal Care Committee.

### Materials and reagents

All chemicals and cell culture reagents were purchased from Sigma-Aldrich (Germany) and Invitrogen (Germany), respectively, unless otherwise specified. The following protein inhibitors and phosphatidylinositols were used in our experiments: Wortmannin (Alexis Biochemicals, USA), PKCζ pseudosubstrate (Biotrend, Switzerland), PKCζ pseudosubstrate inhibitor myristoylated (Calbiochem, Germany), LY294002 (Alexis Biochemicals, USA), Rac1 inhibitor (Calbiochem, Germany), Akt inhibitor X (Calbiochem, Germany), *Clostridium difficile *Toxin A (Calbiochem, Germany), Raf1 kinase inhibitor (Calbiochem, Germany), manumycin A (Calbiochem, Germany), rapamycin (Calbiochem, Germany), phosphatidylinositol-(3,4,5)-P3 (PIP3,4,5) (Cayman chemical, USA), phosphatidylinositol-(3,4)-P2 (PIP3,4) (Cayman chemical, USA), phosphatidylinositol-(4,5)-P2 (PIP4,5) (Calbiochem, Germany).

Rac1 and Cdc42 constitutively active (pcDNA3.1(+)hCdc42G12V and pcDNA3.1(+)hRac1G12V) and inactive (pcDNA3.1(+)hCdc42T17N and pcDNA3.1(+)hRac1T17N) constructs were purchased from UMR (University of Missouri Rolla, USA). Constitutively active Akt1 mutant construct, pUSEamp(+)myr-Akt1, was purchased from Upstate (USA).

PCMV-SPORT6-Pik3r1 and -Prkcz were purchased from Biocat (Heidelberg, Germany).

The following antibodies were used in our analysis: rabbit anti-EGFP antibody, 1:10000 (Molecular Probes, USA), mouse anti-beta-tubulin III, Tuj1, 1:500 (Covance, USA), mouse anti-Tau, 1:1000 (Chemicon, UK), rabbit anti-Tau 1:1000 (Santa Cruz, Germany), mouse anti-MAP2 1:500 (Chemicon, UK), Alexa 488-conjugated anti-rabbit and anti-mouse IgG (Invitrogen GmbH, Germany), anti-mouse and anti-rabbit Cy3 coupled and anti-mouse Cy5 coupled secondary antibody (Jackson Immuno Research Laboratories, USA).

### Preparation of SVZ/RMS culture

SVZ/RMS areas were dissected from coronal sections of P6-10 wild-type mice. All steps of tissue processing were done in 1×Dissection Media (10×DM: 100 mM MgCl_2_, 10 mM kynurenic acid, 100 mM HEPES in 1×Hank's Balanced Salt Solution). Brains were placed in 1×DM media and coronal sections containing the anterior part of the SVZ and posterior part of the RMS were dissected using a blade. Regions around the lateral ventricles were isolated and washed in 1×DM. Dissected SVZ/RMS areas were incubated for 5 min with 30 U of papain (Worthington, USA) and 0.0005% DNase solution, and washed by trypsin inhibitor (Sigma-Aldrich, Germany) with 0.0005% DNAse in Neurobasal Media Supplemented [500 ml of Neurobasal media + 10 ml 50×B27-Supplement + 1.25 ml 200 mM L-Glutamate + 5 ml penicillin/streptomycin (100 U/ml)]. Cells were triturated through a fine tip, counted and plated at appropriate densities in Neurobasal Media with serum [500 ml of Neurobasal media + 50 ml FBS + 10 ml 50×B27-Supplement + 1.25 ml 200 mM L-Glutamate + 5 ml penicillin/streptomycin (100 U/ml)]. After 2 hours Neurobasal Media with serum was changed to Neurobasal Media Supplemented. Half of the media was changed every 4 days.

### Neurite outgrowth assay

Cultured SVZ/RMS cells were treated for one or four days with different protein inhibitors: LY294002 - 30 μM, Wortmannin - 500 nM, PKCζ pseudosubstrate inhibitor - 0.1-0.5 μM, Akt inhibitor X - 500 nM, *Clostridium difficile *Toxin A - 100 ng/ml, Rac1 inhibitor - 50 μM, Raf1 kinase inhibitor - 2 μM, rapamycin - 50 pM; PIP3,4,5 - 10 μM; PIP3,4 - 10 μM; PIP4,5 - 10 μM. For positive apoptosis control inhibitor we used manumycin A (5 μM), an inhibitor of the Ras cell survival pathway [41]. Cells were then fixed and stained with DAPI and anti-Tuj1 antibodies. For treated and untreated samples, the length of the longest neurite of Tuj1-positive cells was determined in 5 circles across the cell growth area (3 samples, number of cells > 100).

To overexpress different genes of the intracellular signaling pathway, cultured SVZ/RMS cells were co-transfected with the pEGFP and the particular gene expression construct. As control we used pCMV-SPORT6 plasmid. Two μl of Lipofectamine 2000 (Invitrogen GmbH, Germany) were mixed with 1 μg of plasmid DNA. The mixture was incubated at room temperature for 30 min and applied to SVZ/RMS cultures. After 1 hour at 37°C, cells were washed and incubated for another hour in Neurobasal media with serum (see recipe above). Subsequently, the cells were washed with Neurobasal Media Supplemented and conditioned media was added back. After 4 days in culture, cells were fixed and stained using anti-Tuj1 and anti-EGFP antibodies. For each gene expression construct, the length of all neurites for double-labeled EGFP and Tuj1-positive cells was calculated (3 samples, number of cells > 100).

The effect of PIPs was examined by intracellular PIP delivery into the cultured SVZ/RMS cells. Four days after PIP delivery, cells were fixed and stained with DAPI and anti-Tuj1 antibodies. The number of Tuj1-positive cells having one or more long neurites was determined (3 samples, n > 100).

### Intracellular delivery of PIPs

Stock solutions of PIPs and neomycin at 1 mM concentration were prepared in HEPES-buffered saline. PIPs were mixed with a carrier (neomycin) to 10 μM each in Neurobasal Media, incubated at room temperature for 10 min, followed by 10 s of bath sonication (SONOREX, Bandelin GmbH & Co. KG, Germany). PIP-carrier containing medium was applied to SVZ/RMS cultures.

### Rac1 and Cdc42 pull down assays

Three million cells from the SVZ/RMS area were plated on 10 cm plates coated with poly-L-lysine. After 3 days in culture, different protein inhibitors were added (concentrations were the same as in neurite outgrowth assay, except of for PKCζ inhibitor - 0.5 μM), and cells were cultured for one additional day. Rac1-GTP and Cdc42-GTP concentrations were analyzed by Rac1 and Cdc42 pull-down kits (Cytoskeleton, USA) according to the manufacturer's recommendations.

### Immunocytochemistry

Cultures were fixed with 4% paraformaldehyde for 1 hour and then blocked in 0.5-1% Triton and 1% normal goat serum. Primary and secondary antibodies were described above. Sections were mounted onto slides with Moviol and subsequently analyzed on an upright fluorescent microscope (Zeiss Axioplan 2).

### Western-blot analysis

For Western-blot analysis protein samples were boiled in SDS gel sample buffer. Denatured proteins were separated by SDS-PAGE, transferred onto PVDF membranes and probed with antibodies. For statistical analysis antibody signals were quantified using ImageJ software and values were normalized to the corresponding β-actin signals. Sample sizes were n ≥ 3 and statistical analysis was performed with paired t-test.

### Analysis of cell death

Cell death in dissociated SVZ/RMS cultures was estimated by adapting a protocol from [42]. Briefly, cultures were incubated for 20 min in 5 μg/ml of propidium iodide, fixed for 4 h in 4% PFA, stained with appropriate antibodies and analyzed on an upright fluorescent microscope (Zeiss Axioplan 2).

### Statistical analysis

The major neurite length and total neurite length were measured using Image J software. The normality of distribution was analyzed by d'Agostino and Shapiro-Wilk tests. We used ANOVA test for multiple comparisons and t-test for pair-wise comparisons. Differences were considered significant at p < 0.05. The graphs show mean ± standard deviation. For all pharmacological analysis at least 3 independent experiments for each condition were used and cell count was performed on 5 randomly picked areas on a coverslip. For all genetic analysis at least 3 independent experiments for each condition were used and at least 100 cells were analyzed.

## Authors' contributions

KK conceived the study, designed and carried out the experiments, drafted the manuscript. HM conceived the study and helped to draft the manuscript. Both authors read and approved the final manuscript.

## Supplementary Material

Additional file 1 **Supplementary online material**. Table S1. *In situ *hybridization signal in SVZ, RMS and olfactory bulb according to the Allen Brain Atlas for the genes selected for *in vitro *analysis. Figure S1. Effect of indicated inhibitors on adhesion and apoptosis of SVZ/RMS cells. Click here for file
