# Supplementary material for: Signaling involved in neurite outgrowth of postnatally born subventricular zone neurons in vitro
Source: BMC Neurosci. 2010 Feb 10;11:18. doi: 10.1186/1471-2202-11-18 (PMC2831042; doi:10.1186/1471-2202-11-18)
Supplement: Additional file 1 — Supplementary online material. Table S1. In situ hybridization signal in SVZ, RMS and olfactory bulb according to the Allen Brain Atlas for the genes selected for in vitro analysis. Figure S1. Effect of indicated inhibitors on adhesion and apoptosis of SVZ/RMS cells. [file 1471-2202-11-18-S1.DOC]

## Supplementary online material

Table S1. *In situ* hybridization signal in SVZ, RMS and olfactory bulb according to the Allen Brain Atlas for the genes selected for *in vitro* analysis.

| Gene name | SVZ | RMS | olfactory bulb |
| --- | --- | --- | --- |
| *Pik3r1* (PI3K) | +++^1^ | +++ | ++ |
| *Akt1* | ++ | +++ | ++ |
| *Prkcz* (PKCζ) | n.s.^2^ | + | + |
| Rac1 | + | +++ | +++ |
| *Cdc42* | +++ | +++ | +++ |

^1^Strength of *in situ* hybridization signal: + - weak, ++ - moderate, +++ - strong. Signal was analyzed on sagittal as well as coronal sections. Strength of the hybridization signal was measured according to the signal intensity of the randomly chosen set of 100 genes.

^2^n.s. – no signal in the area according to the Allen Brain Atlas.


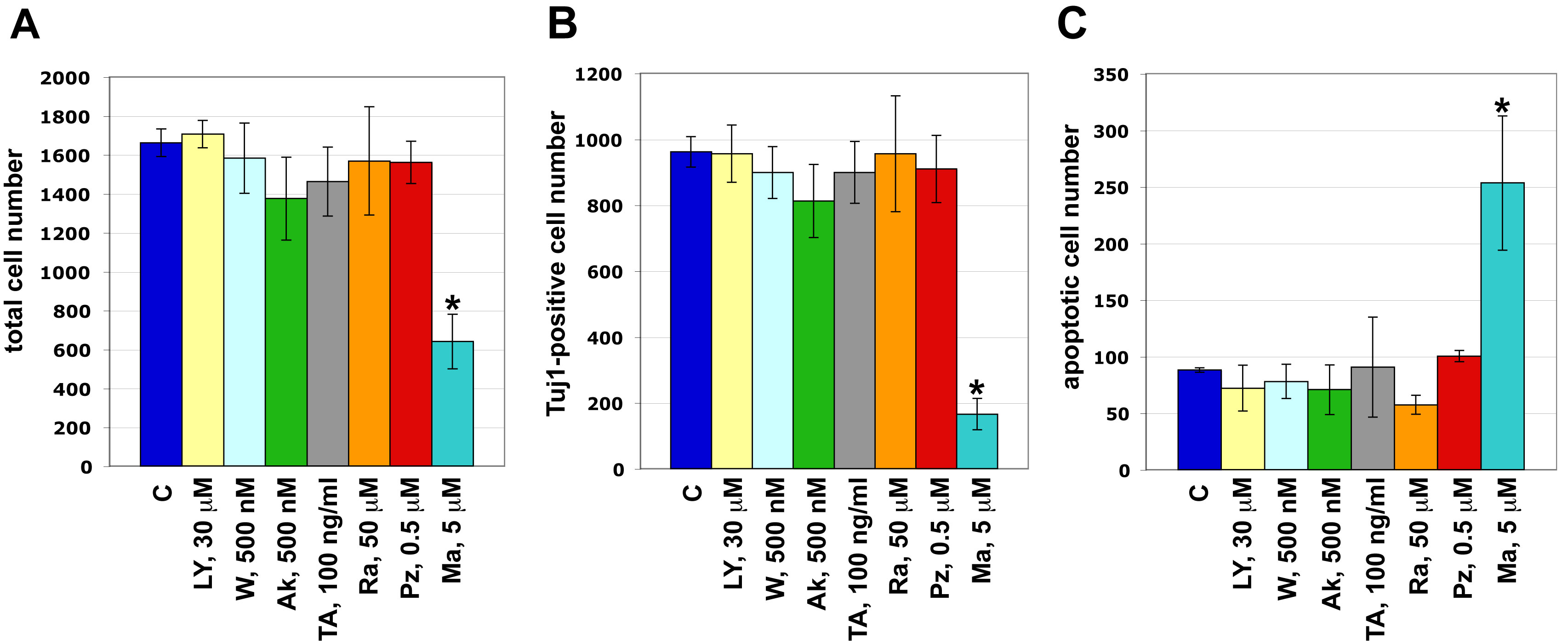


**Figure S1. Effect of indicated inhibitors on adhesion and apoptosis of SVZ/RMS cells.** (A) Total number of cells, DIV1 (* - p<0.001). (B) Number of Tuj1-positive cells (immature neurons) (* - p<0.001). (C) Number of propidium iodine-positive cells (apoptotic cells) (* - p<0.001). Abbreviations: Ak – Akt1 inhibitor, C – control, LY – PI3K inhibitor LY294002, Ma – Ras inhibitor manumycin A, PZ – PKCζ inhibitor, Ra – Rac1 inhibitor, TA – Rho GTPases inhibitor Toxin A of *C. difficile*, Tuj1 – tubulin beta III to label immature neurons, W - PI3K inhibitor wortmannin.
